# Upper facial chorea in Huntington disease

**DOI:** 10.1186/2054-7072-1-7

**Published:** 2014-11-20

**Authors:** Robert Fekete, Joseph Jankovic

**Affiliations:** Department of Neurology, Parkinson Disease Center and Movement Disorders Clinic, Baylor College of Medicine, Houston, Texas USA; Department of Neurology, New York Medical College, Valhalla, NY USA

**Keywords:** Huntington disease, Chorea

## Abstract

To provide a systematic description of component movements of upper facial chorea in Huntington disease, consecutive videos of 25 active patients with confirmed diagnosis were scored on eye opening, eye closing, and procerus/corrugator contractions. Of the 25 patients evaluated, 76% exhibited intermittently widened palpebral fissures associated with frontalis contractions. Brief periods of repetitive but irregular blinking were observed in 16%. 8% had brief spasms of the orbital portion of the orbicularis oculi muscles. In addition, brief contractions of procerus and corrugator supercilii muscles were noted in 52%.

**Electronic supplementary material:**

The online version of this article (doi:10.1186/2054-7072-1-7) contains supplementary material, which is available to authorized users.

## Introduction

In his seminal description of the disorder which now bears his name, George Huntington noted that “the eyelids are kept winking, the brows are corrugated, and then elevated, the nose is screwed first to the one side and then to the other, and the mouth is drawn in various directions” [1, 2]. More detailed descriptive information about facial chorea in Huntington disease (HD) is lacking in modern literature. We have systematically studied involuntary movements in the face of patients with documented HD to further characterize upper facial chorea.

## Materials and methods

Consecutive patients with HD followed in the Movement Disorders Clinic, Baylor College of Medicine over a period of one year were selected for this study. Videos of 25 patients with confirmed diagnosis of HD and their medical records were reviewed and scored on the following scale, adopted from the Jankovic Rating Scale [3]: Eye Closing (0 – 4), Eye Opening (0–4); and Procerus/Corrugator Contractions (0–3) (Table 1). This study was approved by the institutional review board at Baylor College of Medicine. All patients signed written informed consent for videotaping and for the resulting videotapes and associated clinical information to be used for research and publication purposes.

**Table 1:** **Rating scales used for eye closing, eye opening, and procerus/corrugator supercilii contractions**

Score	0	1	2	3	4
**Eye closing**	Rare or absent eyeblinks	Normal rate of blinking	Fast rate of eye blinks	Continuous repetitive blinking	Blepharospasm
**Eye opening**	Eyes partially closed	Normal eye opening	Moderately increased frequency of eye openings	Markedly enlarged palpebral fissure but without eyebrow elevation or frontalis contractions	Markedly enlarged palpebral fissure with marked eyebrow elevation and frontalis contractions
**Procerus**	Absent	Rare	Frequent	Continuous	

## Findings

The demographics and genetic background of our population of 25 HD patients was representative of typical HD patients evaluated in our movement disorders clinic (Table 2). Of the 25 patients evaluated, 19 (76%) exhibited intermittently widened palpebral fissures associated with frontalis contractions; the remaining 6 (24%) manifested elevated rate of eye opening. In the eye closure ratings, 19/25 (76%) had elevated rate of eye blinks, brief periods of repetitive blinking were observed in a distinct group of 4/25 (16%) and another 2/25 (8%) had brief irregular spasms of the orbital portion of the orbicularis oculi muscles (Additional file 1). Random contractions of the procerus and corrugator supercilii muscles were noted in 52% of all patients. This consists of distinct ratings of frequent in 6 (24%), continuous in 4 (16%), and rare in 3 (12%) of patients.

**Table 2:** **Demographic and genetic details of patient cohort**

Age at diagnosis (years)	Average age (years)	% female	CAG repeat length average*	CAG repeat range*	UHDRS total motor score**
50 ± 16	52	48%	44.1	40-65	35 ± 19

*Available for 11/25 patients; others were recorded as CAG repeat positive without actual repeat length.

**Available for 17/25 patients.

## Discussion

In this study of 25 HD patients, upper facial chorea manifested chiefly by brief, irregular widening of palpebral fissures and intermittent, irregular blinking. This phenomenological characterization may be used in the differential diagnosis of facial dyskinesias. Although we acknowledge limitations of this small study, including small sample size and the use of a single rater, we believe that this careful examination of upper face chorea provides useful information about the phenomenology of this movement disorder.

Chorea is characterized by brief, rapid, irregular and random movements that may involve any part of the body. Patients with HD often tend to camouflage the movements by incorporating them into semipurposeful movements (“parakinesia”), which may delay the recognition of chorea and initially hamper the diagnosis. In some cases, facial movements associated with HD may be difficult to differentiate from those observed in patients with other facial dyskinesia such as blepharospasm, tardive dyskinesia, tics, and even hemifacial spasms [4]. Although we did not compare our patients with HD with those of other abnormal movement disorders involving the face, partly because of because of the broad spectrum of phenomenology we believe that we can make some clinical observation that may be helpful in differentiating upper face chorea from other facial dyskinesias. While bilateral orbicularis oculi contractions occur in blepharospasm, the combination of abnormal eye opening and corrugator supercilii contractions in HD should distinguish upper facial movements in these two disorders. Furthermore, the periorbital contractions associated with HD are brief, irregular and random in contrast to more patterned and sustained contractions typically present in patients with blepharospasm. Rare bilateral hemifacial spasm has been reported [4], but the “other Babinski sign” [5] manifested by elevation of the eyebrow due to ipsilateral frontalis contraction and simultaneous orbicularis oculi contraction leading to eye closure, typically seen in the setting of hemifacial spams, has not been seen in HD. Instead, frontalis contractions in HD are associated with eye opening (Additional file 1). Upper facial motor tics pose a diagnostic challenge, especially when there are no associated motor or phonic tics, but the presence of premonitory sensation should assist in distinguishing facial tics from chorea [4]. Of note, rare cases of motor as well as vocal tics with premonitory sensation as presenting signs of HD have been reported [6].Upper facial involvement is rare in tardive dyskinesia, another movement disorder typically involving the face [7].

## Conclusion

In conclusion, the primary aim of study was to draw attention to involvement of upper face in HD and to note that the appropriate recognition of the phenomenology should lead to the correct diagnosis and appropriate treatment [8].

## Electronic supplementary material

Additional file 1: **Video Legend: Upper facial chorea in two patients with HD, illustrating abnormal eye opening, eye closing, and corrugator supercilii contraction.** (MPEG 7 MB)
